# Modern analyses on an historical data set: skull morphology of Italian red squirrel populations

**DOI:** 10.3897/zookeys.368.4691

**Published:** 2014-01-08

**Authors:** Giovanni Amori, Gaetano Aloise, Luca Luiselli

**Affiliations:** 1 CNR – Institute of Ecosystem Studies, viale dell’Università 32, I-00185 Rome, Italy; 2 Natural History Museum and Botanical Garden, Calabria University; 3 Environmental Studies Centre Demetra s.r.l., Rome, Italy

**Keywords:** Morphometrics, red squirrel, Italy, historical dataset

## Abstract

Recent molecular evidence suggests that *Sciurus vulgaris* populations from Calabria (southern Italy) are distinct from those occurring in northern and central Italy. Here, we re-analyzed using multivariate and univariate techniques an historical dataset provided by Cavazza (1913), who documented measurements for the now extinct squirrel population from Campania. Both univariate and multivariate analyses confirmed that the sample from Calabria was homogenous and relatively distinct compared to the rest of the squirrel samples.

## Introduction

The Eurasian red squirrel, *Sciurus vulgaris* Linnaeus, 1758, is characterized by great variability in fur coloration, which led to the description of more than 40 subspecies throughout its wide geographic distribution across the Eurasian continent (Corbet 1978). Currently only 17 of these subspecies are considered valid (Lurz et al. 2005), with the Italian populations being ascribed to three subspecies (Toschi 1965). These Italian subspecies are:

1) *Sciurus vulgaris fuscoater* Altum, 1876 (European form occurring in the Alps and in the northern Apennines), characterized by relatively small size and a strong degree of coat-colour polymorphism both within and between populations;
2) *Sciurus vulgaris italicus* Bonaparte, 1838 (endemic to Central Italy), also characterized by relatively small size, albeit bigger than the previous subspecies. This subspecies shows some degree of coat colour polymorphism, with the dark brown morph dominant in mountainous forests at higher altitudes. The populations of the southern tip of the range are black (subspecies *Sciurus vulgaris alpinus*, *sensu* Costa 1839);
3) *Sciurus vulgaris meridionalis* Lucifero, 1907 (endemic to the most southern Apennines), with uniform fur colour, always having black dorsal fur with grey shades on the sides, a black tail, and a contrasting white belly. It is also the largest Italian subspecies (Wauters and Martinoli 2008).

Although widespread in Italy, this species’ distribution is associated with forested areas, and affected by their fragmentation (Celada et al. 1994, Wauters et al. 1994a, Wauters et al. 1994b, Wauters 1997, Hale et al. 2001). Thus, the European squirrel currently occurs in the whole of the Italian Peninsula with some distribution gaps: the species does not currently occurs in Campania, Apulia and Basilicata (cf. Wauters and Martinoli 2008). However, the squirrel was present in historical times also in the extreme northern part of Campania (i.e. Mt. Somma – Vesuvio) (Costa 1839, Trouessart 1910, Cavazza 1913), where it is now extinct (Capolongo and Caputo 1990, Maio et al. 2000).

Recent molecular data (Grill et al. 2009) revealed the presence of two main mitochondrial phylogroups: (i) a clade comprising the individuals from the region of Calabria in southern Italy belonging to the subspecies *Sciurus vulgaris meridionalis*, and (ii) another including the rest of the Italian populations.

Cavazza (1913) studied morphological variability of Italian populations of *Sciurus vulgaris*, and provided a useful set of skull measurements for squirrels collected throughout Italy. Among various populations, he analyzed specimens from an area where the species is now locally extinct (Campania), which is geographically closer to the populations of the subspecies *Sciurus vulgaris italicus* than to those of *Sciurus vulgaris meridionalis*. Cavazza’s (1913) data are important for evaluating whether the extinct Campanian squirrels were more similar to those currently inhabiting Calabria, or to those typical of central Italian regions.

In this paper, we reanalyzed Cavazza’s original dataset using modern statistical multivariate analyses with the aim to evaluate whether morphometric and genetic data agree with respect to patterns of geographic differentiation in Italian squirrel populations.

## Materials and methods

**Figure 1.**
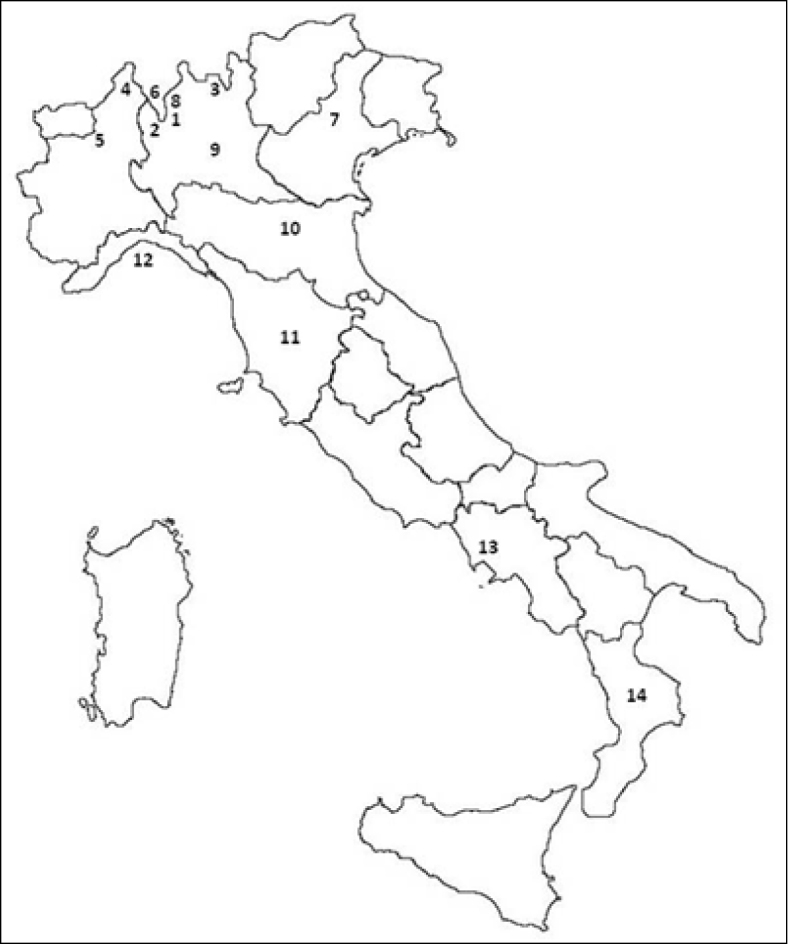
Map of Italy showing the localities where squirrels were collected according to Cavazza (1913). **1** Porlezza **2** Lanzo **3** Central Alps **4** Alpi Piemontesi **5** Biellese **6** Lugano **7** Bassano del Grappa **8** Buggiolo **9** Lombardia **10** Emilia **11** Tuscany **12** Liguria **13** Neapolitan (Campania) **14** Calabria.

We used the data reported in Cavazza (1913) for skull measurements of adults (Table 1). Cavazza (1913) divided specimens into the following groups: (a) Alps, (b) northern and central Italy including Latium and excluding Abruzzi, (c) southern Italy including Abruzzi and Campania, and (d) Calabria. The localities where Cavazza (1913) collected his specimens are reported in Figure 1. Unfortunately, we cannot re-measure specimens from Cavazza’s (1913) paper because several of them have now become lost. Moreover, although it is possible that some of the specimens originarily measured by Cavazza (1913) are still available in private or public collections in Italy, unfortunately there is no labeling indication in Cavazza’s paper for any of his specimens, and this fact impeded us from any further analysis of the vouchers.

**Table 1.** Skull measurements (in mm). OTU = Operational Taxonomic Unit; A = Alps; B = North and Central Italy; C = Abruzzo and Campania; D = Calabria (from Cavazza 1913). For more details see text.

ID	OTU	Skull length	Skull width	Skull height	Mandible length	Interorbital width	Locality	sex
17Alpf	A	50.1	28.5	19.6	27.3	18.3	AlpiCentrali	f
8Apf	A	51.9	29.6	23.4	28.0	19.2	Lanzo	f
7Alpm	A	49.7	28.3	21.0	28.2	18.8	Lanzo	m
12Aplf	A	51.8	30.0	21.0	28.2	19.0	Porlezza	f
7Alpf	A	51.3	29.0	23.3	28.8	18.9	Lanzo	f
3Alpf	A	52.0	29.0	22.0	28.9	20.0	AlpiPiem.	f
9Alpf	A	51.2	29.1	21.3	28.9	18.4	Porlezza	f
5Alpm	A	49.6	29.0	20.0	29.0	19.2	Biellese	m
13Alpm	A	51.5	31.2	21.6	29.0	20.0	Porlezza	m
2Alpf	A	52.6	29.6	22.0	29.0	20.0	AlpiPiem.	f
4Alpf	A	51.8	29.0	21.0	29.0	19.8	Biellese	f
15Alpf	A	52.6	30.6	19.9	29.0	20.0	SopraLugano	f
18Alpf	A	51.7	29.8	21.0	29.0	18.2	AlpiCentrali	f
25Alpf	A	50.1	28.7	21.0	29.0	19.0	Cadore	f
26Alpf	A	51.2	29.6	22.0	29.0	19.4	Cadore	f
9Alpm	A	51.8	30.6	21.0	29.1	19.5	Lanzo	m
16Alpf	A	55.0	31.2	21.0	29.1	18.6	AlpiCentrali	f
1Alpf	A	53.0	30.8	22.0	29.2	21.0	AlpiPiem.	f
5Alpf	A	50.8	28.9	22.6	29.2	18.7	Lanzo	f
13Alpf	A	55.9	31.0	22.6	29.2	20.2	Buggiolo	f
21Alpf	A	53.0	30.0	21.2	29.2	19.0	AlpiCentrali	f
2Alpm	A	57.3	32.0	21.0	29.3	20.0	AlpiPiem.	m
10Alpm	A	52.2	31.0	21.0	29.3	20.0	Lanzo	m
14Alpm	A	49.9	27.8	20.8	29.3	18.3	Porlezza	m
6Alpf	A	52.0	29.8	23.0	29.3	19.1	Lanzo	f
14Alpf	A	52.6	30.3	21.8	29.3	21.0	SopraLugano	f
20Alpf	A	51.8	29.9	21.2	29.3	19.0	AlpiCentrali	f
19Alpf	A	52.0	30.0	21.3	29.4	19.5	AlpiCentrali	f
12Alpm	A	52.0	30.2	21.6	29.5	20.0	Lanzo	m
3Alpm	A	50.0	29.2	20.4	29.6	19.2	AlpiPiem.	m
6Alpm	A	52.7	30.3	22.2	29.8	19.8	Lanzo	m
1Alpm	A	53.0	32.2	21.2	30.0	20.7	AlpiPiem.	m
18Alpm	A	52.8	32.0	21.0	30.0	20.0	SopraLugano	m
4Alpm	A	53.0	33.0	21.3	31.0	20.0	Biellese	m
26Alpm	B	51.5	30.3	21.0	28.9	19.3	Cadore	m
25Alpm	B	51.6	30.8	20.8	29.0	19.0	Cadore	m
1Lomm	B	52.2	29.4	22.6	29.0	18.8	Lombardia	m
2Emim	B	52.6	29.0	22.2	29.0	19.4	Emilia	m
3Emif	B	50.1	29.0	21.8	29.0	18.6	Emilia	f
9Tosf	B	53.2	30.2	22.1	29.0	18.8	Toscana	f
10Tosf	B	52.8	29.7	22.9	29.0	18.0	Toscana	f
4Emif	B	51.1	29.3	22.0	29.1	18.9	Emilia	f
5Emif	B	52.0	29.0	22.0	29.1	18.7	Emilia	f
11Tosm	B	52.0	30.0	22.0	29.2	18.6	Toscana	m
1Ligf	B	51.2	28.3	22.0	29.2	18.3	Liguria	f
6Emif	B	52.2	29.1	22.3	29.3	18.6	Emilia	f
7Emim	B	52.7	30.1	22.3	29.5	19.0	Emilia	m
3Emim	B	53.7	29.6	23.0	29.6	19.9	Emilia	m
8Emim	B	52.7	30.2	22.2	29.6	18.9	Emilia	m
10Tosm	B	53.2	30.1	22.0	29.6	19.0	Toscana	m
9mim	B	52.7	30.3	22.2	29.8	19.0	Emilia	m
1Alpm	B	52.9	30.1	21.3	30.0	19.8	AlpiCentrali	m
20Alpm	B	52.3	30.1	20.6	30.0	18.9	AlpiCentrali	m
22Alpm	B	50.0	31.0	22.0	30.0	18.8	AlpiCentrali	m
6Emim	B	53.5	34.3	22.1	30.0	18.0	Emilia	m
12Tosf	B	53.0	30.8	22.9	30.0	18.3	Toscana	f
13Tosf	B	52.3	30.3	22.8	30.0	18.7	Toscana	f
18Tosm	B	52.2	31.0	22.3	30.2	18.7	Toscana	m
21Alpm	B	53.1	32.0	21.2	30.3	19.7	AlpiCentrali	m
17Tosm	B	53.0	32.0	22.0	30.3	18.0	Toscana	m
13Tosm	B	52.0	30.0	20.8	30.6	18.2	Toscana	m
11Tosf	B	52.0	31.5	23.0	31.0	18.7	Toscana	f
12Tosm	B	55.0	31.9	21.0	31.2	19.0	Toscana	m
3Napf	C	52.3	29.7	24.1	28.9	18.6	Napoletano	f
4Napf	C	54.6	29.9	25.0	29.0	19.0	Napoletano	f
3Napm	C	52.8	28.9	22.9	29.2	19.0	Napoletano	m
2Napf	C	54.3	29.8	24.9	29.4	18.9	Napoletano	f
4Napm	C	55.0	29.8	22.8	29.5	19.6	Napoletano	m
2Napm	C	55.2	31.3	24.0	30.0	20.0	Napoletano	m
2Calf	D	56.3	33.6	22.7	31.8	19.1	Calabria	f
3Calm	D	56.0	33.9	22.4	32.2	19.0	Calabria	f
1Calm	D	56.0	33.5	22.6	33.9	20.7	Calabria	f
1Calf	D	57.2	33.4	22.8	33.9	19.2	Calabria	f
2Calm	D	54.5	32.9	22.3	34.1	20.2	Calabria	f

Univariate measurements were log-transformed in order to achieve normality and then compared across groups by one-way Analysis of Variance (ANOVA). In this analysis, the same four groups as defined by Cavazza (1913) were used.

Specimens were divided into four Operational Taxonomic Units (hereby OTUs), according to their geographical provenance and corresponding to the Italian subspecies. These four OTUs followed exactly the subdivisions made by Cavazza (1913). We performed a cluster analysis in order to show dissimilarities among all of Cavazza’s (1913) specimens in terms of their skull measurements. Skull measurements were log-transformed prior to analysis. Dendrograms were prepared using the single linkage as the algorithm, with Euclidean distances. This method was used because it provided the highest cophenetic index. In the single linkage (nearest neighbour), the clusters are joined based on the smallest distance between the two groups. Branch support was calculated with 10,000 bootstrap replicates. We also used neighbour joining clustering (Saitou and Nei 1987), which is an alternative method for hierarchical cluster analysis. In contrast with ultrametric methods (like the Unweighted Pair Group Method with Arithmetic Mean, UPGMA), two branches from the same internal node do not need to have equal branch lengths. A phylogram (unrooted dendrogram with proportional branch lengths) is given in this paper.

We studied the dispersion of specimens in multivariate space with Principal Components Analysis (PCA) using the covariance matrix (Davis 1986, Harper 1999) (PC1 scores serve as a proxy for size, while the other PCs capture shape variation).

## Results

The original dataset reported by Cavazza (1913) is summarized in Table 1. Mean and standard deviations for each measurement considered are reported in Table 2 with all specimens pooled, and in Table 3 with samples divided into OTUs. Using the same categories as in Cavazza (1913), there were among-group statistical differences for skull length (one-way ANOVA F_3,70_ = 14.76, P < 0.00001), skull width (F_3,70_ = 13.50, P < 0.00001), skull height (F_3,70_ = 18.93, P < 0.00001), and mandible length (F_3,70_ = 56.83, P < 0.00001), but not for interorbital length (F_3,70_ = 1.92, P < 0.133). Post-hoc Tukey HSD tests revealed that Calabria specimens differed significantly from every other group for mandible length (all P < 0.01), and for skull width (all P < 0.001). For skull length, Calabria specimens differed from Alpine and central Italian specimens (all P < 0.01) but not from Campania specimens (P = 0.088). For skull height, they differed from Campania (P = 0.024) and Alpine specimens (P = 0.018) but not from central Italian specimens (P = 0.43). Principal component scores indicated that there were significant statistical shape differences among the four populational groups (one-way ANOVA: F_3,70_= 30.362, P < 0.0001), and a Tukey HSD post-hoc test revealed that (i) the Calabria population differed significantly from all the others (at least, P < 0.000154), (ii) the Campania population significantly differed, other than from Calabria specimens, also from Alpine specimens (P = 0.022) but not from central Italian specimens (P = 0.470).

**Table 2.** Mean and dispersion measures of the five skull variables analyzed in this study (original dataset from Cavazza (1913), for all sampled specimens pooled together.

	Mean (S.D.)	Range
Skull length	52.61 (1.70)	49.6–57.3
Skull width	30.37 (1.41)	27.8–34.3
Skull height	21.92 (1.04)	19.6–25.0
Mandible length	29.63 (1.18)	27.3–34.1
Interorbital length	19.21 (0.70)	18.0–21.0

**Table 3.** Mean and dispersion measures of the five skull variables analyzed in this study (original dataset from Cavazza (1913), with all sampled specimens divided by OTU. Symbols: A = Alps; B = North and Central Italy; C = Abruzzo and Campania; D = Calabria.

	Mean	SD
A (n = 34)		
Skull length	52.05	1.64
Skull width	30.03	1.19
Skull height	21.42	0.88
Mandible length	29.12	0.62
Interorbital length	19.46	0.74
B (n = 29)		
Skull length	52.37	1.02
Skull width	30.32	1.20
Skull height	21.97	0.68
Mandible length	29.67	0.63
Interorbital length	18.22	0.49
C (n = 6)		
Skull length	54.03	1.20
Skull width	29.90	0.77
Skull height	23.95	0.94
Mandible length	29.33	0.39
Interorbital length	19.18	0.51
D (n = 5)		
Skull length	56.00	0.97
Skull width	33.46	0.36
Skull height	22.56	0.20
Mandible length	33.18	1.08
Interorbital length	19.64	0.76

**Figure 2.**
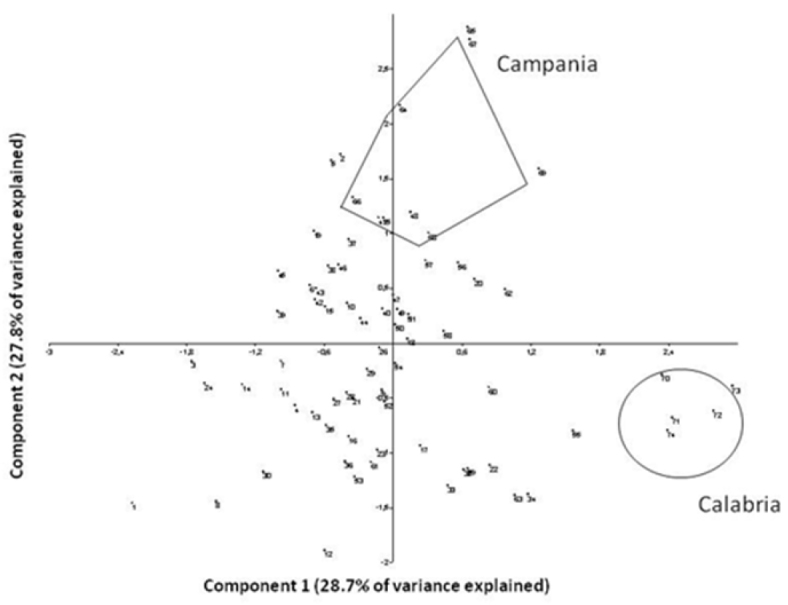
PCA of skull measurements (VARIMAX rotation applied) based on Cavazza’s (1913) dataset. Eigenvalues: component 1 = 2.559; component 2 = 1.099.

**Figure 3.**
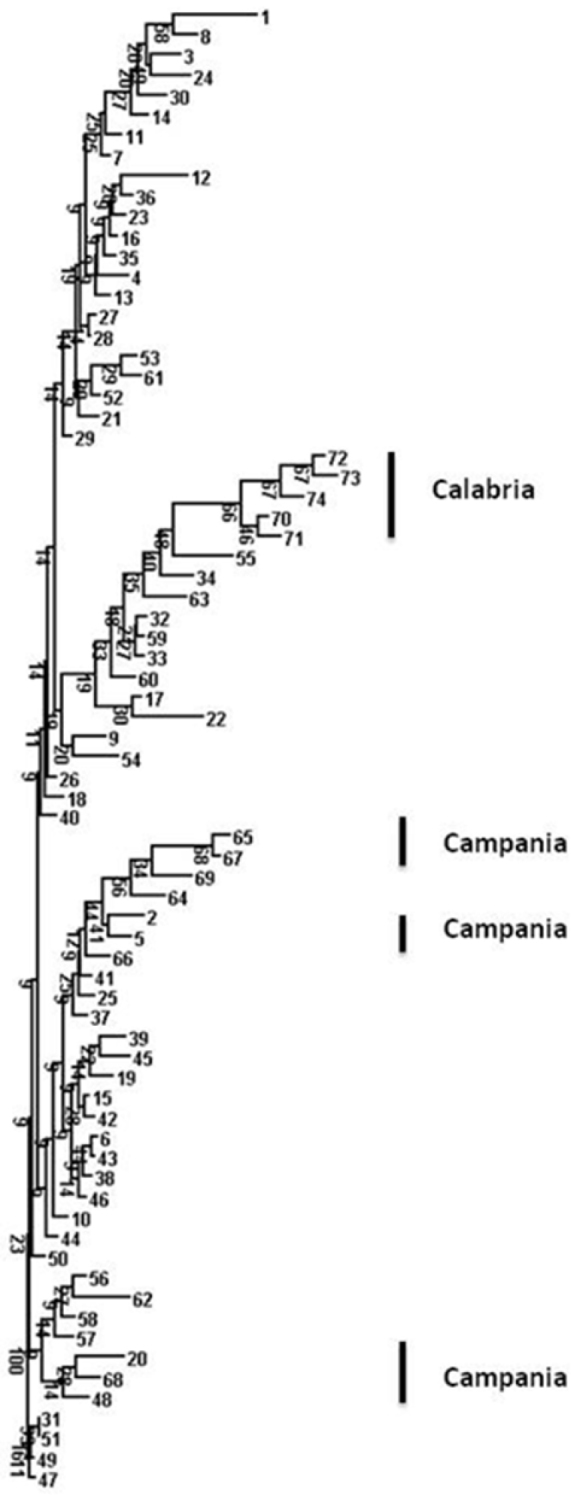
Neighbor joining dendrogram of skull measurements (with 10,000 bootstraps) based on Cavazza’s (1913) dataset.

Both sets of multivariate analyses revealed that the sample from Calabria was homogenous and relatively distinct compared to the rest of the squirrel samples (Figures 2 and 3). In the PCA (variance explained by the first two axes: 56.5%; with axis 1 explaining 28.7% and axis 2 explaining 27.8% of the total variance; see Table 4 for the loadings) there was a trend suggesting clinal variation from the Alps to Campania, with Calabria specimens, while distinct, being more similar to those of Campania than to those of northern Italy (Figure 2). The Campania group showed less variance (Levene’s test; F = 6.67, P < 0.03) compared to the rest of the central and northern Italian samples in the PCA than in the neighbor joining analysis (Figure 3).

**Table 4.** Loadings of the PCA as in Figure 2.

	Component 1	Component 2
Skull length	0.876	0.154
Skull width	0.882	-0.159
Skull height	0.341	0.836
Mandible length	0.842	-0.034
Interorbital length	0.432	-0.684

## Discussion

Both multivariate and univariate tests identified some morphometric differentiation among different squirrel populations that were previously highlighted by the molecular results of Grill et al. (2009). That is: the populations from Calabria differed from the others morphologically (this study) and genetically (D-Loop: Mean genetic distance between groups: 6%, within group: 2%; see Grill et al. 2009). Our analyses also suggest that the currently extinct population from Campania belonged to a central Italian grouping. It may be that patterns of craniometric variation in Italian red squirrels represent a clinal size trend within a formerly contiguous population once occurring from the Alps south to Campania, and, with expectations fitting Bergmann’s rule (e.g., Freckleton et al. 2003; Blackburn and Hawkins 2004).

On the other hand, Calabria specimens do appear to be quite distinct from the rest of the Italian squirrels in size (Figure 2), though we note that our analyses involve quite small sample sizes (Cardini and Elton 2007). Notably, Calabria populations occur mainly at relatively high altitudes, closely linked to that of extensive high-altitude mixed forest dominated by the native Calabrian black pine *Pinus laricio* (Cagnin et al. 2000, Rima et al. 2010) and they are characterized both by large size and monomorphic color fur. Overall, our study could neither substantiate nor reject the hypothesis that *Sciurus vulgaris meridionalis* is a full species, as previously suggested by Gippoliti (2013). However, some morphological differentiation is certainly evident also with respect to the Campania extinct population (this study), and remarkable genetic differences are found between Calabria populations and all the remaining European populations (Grill et al. 2009). Indeed, the majority of individuals analyzed by Grill et al. (2009) formed one monophyletic clade without particular differentiation, whereas Calabrian squirrels were clearly separate. The Calabrian lineage appears to have experienced a different history from the rest of European squirrels probably due to the fact that it became isolated after glaciations and never reconnected to Central Italian populations (Grill et al. 2009). It should be stressed, however, that the sample sizes available for Campania and Calabria were too small to make any firm conclusions.

Our approach in this paper highlights the lasting value of historical publications on biodiversity, especially when they present data on populations which are now extinct. These often overlooked publications – such as Cavazza’s, published in Italian in a regional journal – can be important sources of data that can be re-analysed, for renewed insight, using modern statistical tools.
